# Larvicidal efficacy of *Cryptomeria japonica* leaf essential oils against *Anopheles gambiae*

**DOI:** 10.1186/1756-3305-7-426

**Published:** 2014-09-04

**Authors:** France P Mdoe, Sen-Sung Cheng, Lucile Lyaruu, Gamba Nkwengulila, Shang-Tzen Chang, Eliningaya J Kweka

**Affiliations:** Department of Zoology and Wildlife Conservation, College of Natural and Applied Sciences, University of Dar-es-salaam, P.O. Box 35165, Dar-es-salaam, Tanzania; Experimental Forest, National Taiwan University, Nan-Tou, 557 Taiwan; Division of Livestock and Human Diseases Vector Control, Tropical Pesticides Research Institute, Ngaramtoni, Off Nairobi Road, P.O. Box 3024, Arusha, Tanzania; School of Forestry and Resource Conservation, National Taiwan University, No. 1, Sec.4, Roosevelt Road, Taipei, 106 Taiwan; Department of Medical Parasitology and Entomology, Catholic University of Health and Allied Sciences, P.O. Box 1464, Mwanza, Tanzania

**Keywords:** *Cryptomeria japonica*, Larvicidal efficacy, *Anopheles gambiae* s.s, Mortality, Essential oil

## Abstract

**Background:**

Alternative insecticidal compounds with mortality effect against mosquito life cycle stages are currently needed. The compounds should be biodegradable and nontoxic to non-targeted insects. Plant based larvicides provide effective control of vector populations. This study explored *Cryptomeria japonica* leaf essential oil larvicidal potency against *Anopheles gambiae* sensu stricto.

**Methods:**

Essential oils (12.5 to 200 μg/mL) extracted from *C. japonica* leaves were evaluated against *An. gambiae* s.s. larvae in both the laboratory and semi field in 6 replicates for each dose. Larval mortality readings were taken at 12, 24, 48, and 72 h post treatment.

**Results:**

*C. japonica* leaf essential oil yield was 17.06 ± 0.56 mL/kg and 1.60 ± 0.33% (w/w). GC-FID and GC-MS analyses revealed 22 constituents. Essential oil was more effective against *An. gambiae* s.s. larvae in the laboratory than in semi field trials. Mortality increased with increasing dosages (12.5 to 200 μg/mL) in the laboratory (31.75 to 100%) and semi field trials (17.75 to 99.5%), respectively. The LC_50_ value ranged from 5.55 to 63.92 μg/mL in the laboratory, and 8.22 to 134.84 μg/mL in semi field conditions, LC_90_ value ranged from 41.34 to 205.93 μg/mL in the laboratory and 50.92 to 213.11 μg/mL in semi field conditions.

**Conclusion:**

This study has demonstrated the potential of *C. japonica* leaf essential oil to cause mortality effects to *An. gambiae* s. s. larval populations, however, further studies need to be conducted under field conditions and also with individual active compounds of *C. japonica* essential oil*.*

## Background

*An. gambiae* s.s. is the most effective and efficient vector of the most lethal malaria parasite species (*Plasmodium falciparum*) that predominates Africa, a region where almost 90% of worldwide malaria deaths occur [1, 2]. The transmission of malaria is through a bite by an infected female mosquito [3]. Some deliberate interventions are being implemented to get rid of human vector contact, in Africa, insecticide treated bed nets (ITNs) and indoor residual spraying (IRS) are the key interventions [2]. However, the on-going interventions are threatened by the emergence and spread of resistance genes among mosquito species populations to major insecticides classes such as pyrethroids as well as carbamates and organophosphates to a lesser extent [4]. Therefore, targeting vector mosquitoes at the larval stage is the best alternative since larvae are relatively immobile and confined within a given geographical area, cannot change behaviour to escape the effects of insecticides and thus become more vulnerable as compared to adult mosquitoes [5]. Nevertheless, malaria vector control using synthetic insecticides is also challenged by the insecticidal effects of non-targeted organisms and environmental pollution [6].

Plants constitute a rich source of active and effective compounds that are biodegradable and have traditionally been used to control mosquitoes [7–9]. The complex and variable mixtures of bioactive compounds with different modes of action, offered by plants, may lessen the chance of resistance in mosquito populations. The Japanese cedar (*Cryptomeria japonica* D. Don) leaf essential oil has revealed excellent larvicidal efficacy against *Aedes aegypti* and *Ae. albopictus*, furthermore, the major constituents of the leaf essential oil were effective and displayed various degrees of efficacy when tested individually against larvae of both *Ae. aegypti* and *Ae. albopictus* [10]. Kannathasan and others pointed out that, vector resistance against plant-based insecticides is not yet reported at the moment [11]. Therefore, searching for plant-based pesticides is of paramount importance in vector control and in overcoming the prevailing vector resistance challenges. This study focused on evaluation of *C. japonica* leaf essential oil efficacy against *An. gambiae* s.s. larvae in the struggle to develop an effective, environmentally friendly larvicide and target specific plant-based pesticides that human malaria vector mosquitoes have not developed resistance to.

## Methods

### Plant material

Fresh mature leaves of 28-year-old Japanese cedars (*C. japonica* D. Don) were collected in August 2009 from the Experimental Forest of the National Taiwan University located in the Nantou County in Central Taiwan (longitude: 120°57’56.51”E; latitude: 24°00’46”N; elevation: 600 m). A voucher specimen (CJL006) has been deposited with the Laboratory of Wood Chemistry (School of Forestry and Resource Conservation, National Taiwan University).

### Isolation of essential oils

Fresh mature leaves (200 g) of *C. japonica* were subjected to hydrodistillation (HD) using a *Clevenger*-type apparatus [12] for 6 hrs. Their oil content in percentage (w/w) and mL/kg were determined on the basis of the leaf dry weight. Each test was replicated three times and the essential oils were stored in dark vials at 4°C until further analysis.

### GC-FID analysis

The *C. japonica* leaf essential oils obtained by the two different extraction methods were analyzed with a Finnigan Trace GC apparatus equipped with a flame ionization detector (FID) and a DB-5 cap. Column (30 m × 0.25 mm i.d., film thickness 0.25 μm). The oven temperature was programmed isothermally at 50°C for 2 min and was then increased from 50°C to 250°C at 5°C/min; injector temperature, 270°C; carrier gas, He(1 mL/min). The samples (1 μL) were injected neat with a 1:60 split ratio.

### GC-MS analysis

The compositions of leaf essential oils were analyzed with a Finnigan Trace GC–Polaris Q mass instrument (Finnigan-Spectronex) equipped with a fused-silica column (30 m × 0.25 mm i.d.) coated with DB-5 ms (df = 0.25 μm). The oven temperature was programmed as described above; injector temperature, 270°C; ion-source temperature, 230°C; carrier gas, He (1 mL/min). The samples (1 μL) were injected neat with a 1:60 split ratio, and the mass spectra (ionization energy, 70 eV) were recorded at 1 scan/s over the *m/z* range 50–400 amu.

The percentage composition was computed by integrating the GC-FID peak area. The identification of the essential oil constituents was based on the comparison of their Kovats index (KI) determined relative to the retention times (t_R_) of a series of *n*-alkanes (C_9_-C_24_) on the non-polar DB-5 ms column with those provided in the literature [13] and their mass spectra with those obtained with authentic standards available in the authors’ laboratory and those of the NIST/NBS and Wiley spectra libraries.

### Mosquito larvae

Larvae of *Anopheles gambiae* sensu stricto used in both laboratory and semi field trials were obtained from a laboratory colony at the insectary of Tropical Pesticide Research Institute (TPRI). Rearing of mosquito larvae in the laboratories was as described in other protocols [14]. The laboratory colony was maintained at photo phase of 12 L: 12D with temperature 27 ± 2°C and relative humidity of 78 ± 2%. In both trials, third instar larvae were used as recommended by The World Health Organisation (WHO) protocol [15]. Tetramin fish food was used to feed the larvae.

### Laboratory larvae bioassay

A test solution was prepared by dissolving 2 mL of emulsifier, dimethyl sulfoxide (DMSO), containing test essential oil in 98 mL normal laboratory larvae rearing water in plastic bowls of 100 mL capacity, which was shaken gently to ensure homogeneity followed by serial dilution to obtain 200, 100, 50, 25, and 12.5 μg/mL dosage as described by WHO larvae bioassay protocol [15]. The experiment was set for six replicates in each dosage and two controls; one containing normal laboratory larvae rearing water and the other one 1% aqueous solution of DMSO so as to assess its effect against larvae. Twenty third instar larvae were introduced in each replica and controls for larvicidal assay. Larvae were starved in both treatments and controls during the experiment. Mortality was observed and recorded at intervals of 12, 24, 48, and 72 h post treatment, both dead and moribund larvae were counted as dead.

### Semi field larvae bioassay

Similar dosages to laboratory larvae bioassays were evaluated in semi field larvae bioassays. The structures in the semi field environment were as described in other studies [16]. The evaluation in the semi field environment adhered to the WHO protocol [15]. The dosages were replicated six times and their two controls; one containing normal laboratory larval rearing water and the other one 0.5% aqueous solution of DMSO were set. Twenty third instar larvae were introduced in each treatment and controls for larvicidal assay, then similar procedures to laboratory larvae bioassay were followed to accomplish the semi field larval bioassay.

### Statistical analysis

To determine whether there was a statistically significant effect of *C. japonica* leaf essential oil against third instar larvae of *An. gambiae* s.s., Scheffe’s multiple comparison procedure of SAS was used to analyse the percentage mortality and the results were expressed as mean ± SD. Statistically significant results were those with *P* < 0.05. Essential oil activity and toxicity reported as lethal concentration (in μg/mL), which caused 50 and 90% larval mortality were presented as LC_50_ and LC_90_ respectively, at 12, 24, 48 and 72 h recording intervals.

## Results

### Yield and chemical composition of essential oil

The essential oil yield from *C. japonica* leaves was 17.06 ± 0.56 mL/kg with composition of 1.60 ± 0.33% (w/w) based on dry weight, the values are mean ± standard deviation. Twenty two compounds were identified by GC-MS chemical analysis representing about 98.09% of the essential oils, kau-16-rene being the most abundant with 23.29% followed by β-elemol with 18.29% composition, the remaining 20 chemicals range from 0.23-8.23% composition. All identified compounds were categorised into five groups with their relative abundances starting from the largest; oxygenated sesquiterpenes (39.01%), monoterpene hydrocarbons (28.91%), diterpene hydrocarbons (23.29%), oxygenated monoterpenes (6.47%) and sesquiterpenes hydrocarbons (0.41%).

### Laboratory larval bioassay

**Figure 1:**
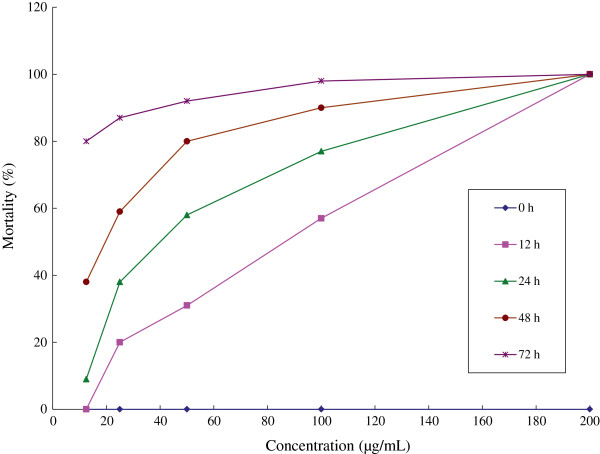
***Anopheles gambiae*** **s.s. larval mortality in the laboratory using different concentrations of** ***Cryptomeria japonica*** **at different time intervals.**

There was a remarkable increase in mortality (31.75 to 100%) when dosages were increased (12.5 to 200 μg/mL), likewise with time (12 to 72 h) mortality increased consistently (41 ± 5.0% to 91.4 ± 12.5%) (Table 1). Furthermore LC_50_ and LC_90_ values were found to be 63.92 and 205.93 μg/mL (after 12 h); 40.90 and 143.42 μg/mL (after 24 h); 17.56 and 104.04 μg/mL (after 48 h); 5.55 and 41.34 μg/mL (after 72 h), respectively (Table 1). Figure 1 shows trends of mortality increase with increased concentration at different mortality recording intervals. Controls did not reveal the effect on larvae. Therefore, data pertaining to larvicidal activity of *C. japonica* leaf essential oil against third instar larvae of *An. gambiae* s.s were statistically significant (*P* < 0.0001) based on both dosages and time (R^2^ = 0.97).

**Table 1:** **The average mortality, LC**_**50**_ **and LC**_**90**_ **of** ***C. japonica*** **leaves essential oil against** ***An. gambiae*** **s.s. larvae in laboratory conditions**

	200	100	50	25	12.5	Equation	R ^2^	LC _50_	LC _90_
0 h	0	0	0	0	0				
12 h	100	57	31	20	0	y = 34.192Ln(x) - 92.159	0.9429	63.92	205.93
24 h	100	77	58	38	9	y = 31.884Ln(x) - 68.329	0.9941	40.90	143.42
48 h	100	90	80	59	38	y = 22.362Ln(x) - 14.08	0.9659	17.56	105.04
72 h	100	98	92	87	80	y = 7.3577Ln(x) + 62.616	0.9734	5.55	41.34

### Semi field larval bioassay

**Figure 2:**
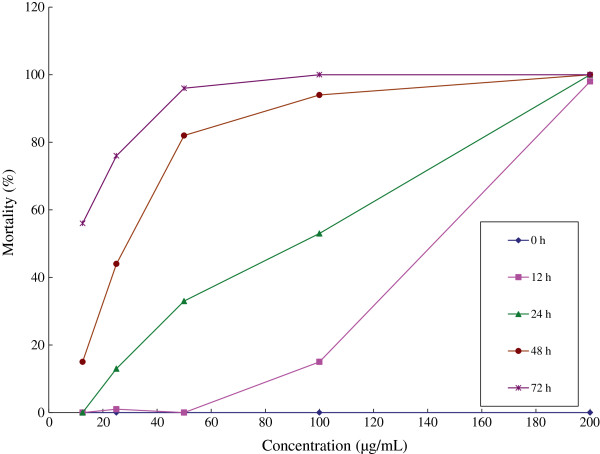
***Anopheles gambiae*** **s.s. larval mortality in a semifield environment using different concentrations of** ***Cryptomeria japonica*** **at different time intervals.**

Mortality increased (17.75 to 99.5%) with increased dosages (12.5 to 200 μg/mL), it was also found that at each successive recording interval (12 to 72 h), mortality increased (22.80 to 85.60%) considerably (Table 1). LC_50_ and LC_90_ values (Table 2) were determined at different time recording intervals. The results indicated larvicidal effect of leaf essential oil at 12 h (LC_50_ = 134.84 μg/mL; LC_90_ = 213. 11 μg/mL), 24 h (LC_50_ = 67.13 μg/mL; LC_90_ = 193.78 μg/mL), 48 h (LC_50_ = 29.27 μg/mL; LC_90_ = 103.21 μg/mL) and 72 h (LC_50_ = 8.22 μg/mL; LC_90_ = 50.92 μg/mL) (Table 2). There was no conspicuous effect against larvae in the controls. The mortality trend based on dosage and time are described (Figure 2). Data recorded showed that leaf essential oil examined in semi field condition exhibited noticeable larvicidal performance.

**Table 2:** **The average mortality, LC**_**50**_ **and LC**_**90**_ **of** ***C. japonica*** **leaf essential oil against** ***An. gambiae*** **s.s. in semi field conditions**

	200	100	50	25	12.5	Equation	R ^2^	LC _50_	LC _90_
0 h	0	0	0	0	0				
12 h	98	15	0	1	0	y = 0.6786x - 41.5	0.964	134.84	213.11
24 h	100	53	33	13	0	y = 34.625Ln(x) - 95.653	0.9371	67.13	193.78
48 h	100	94	82	44	15	y = 31.739Ln(x) - 57.165	0.9174	29.27	103.21
72 h	100	100	96	76	56	y = 21.929Ln(x) + 3.8134	0.9377	8.22	50.92

## Discussion

Insecticides of plant origin are the focus in the struggle to combat developing insect resistance to synthetic insecticides, like DDT, and the associated challenges such as residual problems in the environment and effect on non-target organisms. In contrast, insecticides of plant origin do not render residual problems to the environment, do not harm non-targeted organisms and are still capable of suppressing pest populations. Although a number of compounds of plant origin are currently reported [10, 17], need for the discovery of more effective plant products is of paramount importance so as to improve insecticide formulation and develop environmentally acceptable insecticides in order to replace conventional insecticides in the control of mosquitoes [18].

In this study, leaf essential oil from 28-year-old Japanese cedars (*C. japonica* D. Don) was explored for its composition and larvicidal performance. Twenty-two compounds were identified by GC-MS chemical analysis representing about 98.09% of the essential oil. Kau-16-rene was the most abundant with 23.29% followed by β-elemol with 18.29% composition (Figure 2). In contrary to the findings of this study, Wang and others identified β-elemol (18.22%) as a dominant compound [17]; Shieh and others identified β-eudesmol (14.67%), α-eudesmol (14.67%) and β-elemol (11.62%) as major compounds in *C. japonica* leaf essential oil [19]. This discrepancy might be due to tree age difference, season of sample collection or other factors [10, 20].

The leaf essential oil of *C. japonica* was found to exhibit great larvicidal performance against third instar larvae of malaria vectors, *An. gambiae* s.s. Data obtained revealed an increase in larvae mortality with increased concentration of the essential oil solution. Mortality in laboratory conditions increased from 31.75% to 100% at dosage increase of 12.5 μg/mL to 200 μg/mL respectively. The LC_50_ and LC_90_ values were 63.92 and 205.93 μg/mL (at 12 h); 40.90 and 143.42 μg/mL (at 24 h); 17.56 and 105.04 μg/mL (at 48 h); 5.55 and 41.34 μg/mL (at 72 h). In semi field conditions, mortality increased from 17.5% to 99.5% at a similar dosage increase, the LC_50_ and LC_90_ values were 134.84 and 213.11 μg/mL (at 12 h); 67.13 and 193 μg/mL (at 24 h); 29.27 and 103.21 μg/mL (at 48 h); 8.22 and 50.92 μg/mL (at 72 h). Therefore, mortality in the laboratory was significantly higher than in semi field conditions, this might be attributed to the exposure of larvicides under sunlight, which might have broken it down into nontoxic products [16]. To get rid of such environmental challenges, botanical larvicides need a continued application for its effective control of mosquito larvae [21]. Essential oil activity may be influenced by the presence of major constituents like kau-16-rene (23.29%) and β-elemol (18.29%), however, [22] reported that minor compounds can also be responsible for larvicidal performance.

Previous studies have revealed the mode of action of *C. japonica* leaf essential oil and that it provides inhibitory performance against *Aedes aegypti* and *Ae. albopictus* larval development hormone [10], therefore, increased concentration of essential oil offered more growth inhibition and subsequently higher larval mortality which was revealed using *An. gambiae* s.s. larvae in this study.

## Conclusions

The findings of this study have shown high larval mortality induced by *C. japonica* leaf essential oil against *An.gambiae* senso stricto, a main malaria vector. However, further studies are needed to assess the effect of individual compounds in both laboratory and semi field conditions on larval mortality and the mechanisms involved.

## Abbreviations

GC-MS: Gas chromatography–mass spectrometry
DDT: Dichlorodiphenyltrichloroethane
DMSO: Dimethyl sulfoxide
WHO: World health organisation
KI: Kovats index
GC-FID: Gas chromatography with flame ionization detector
LC: Lethal concentration.
